# Chinese Foot Binding

**Published:** 1924-06

**Authors:** 


### CHINESE FOOT-BINDING.

The origin of foot-binding in China is lost in antiquity, but, contrary to the general impression, the custom is not widespread throughout the country. In the North it seems never to have been adopted. Certain cities have always been exempt, in spite of prevalence in the surrounding country. Nevertheless, the ill effects of foot-binding are still frequently seen in Chinese hospital practice, and Mr. H. C. Patrick, surgeon to the Chinese Hospital, Shanghai, gives a graphic account of these matters in the *Glasgow Medical Journal*. Until knitting machines reached China, the actual covering of the foot was always a cotton bandage. From the little girl's earliest days the bandage is pulled tighter and tighter, so that not only is the ball of the big toe forced backwards until it touches the heel, but the smaller toes are often turned in under the natural sole. In extreme cases this compression is carried to such a point that gangrene sets in and some of the toes, or even the whole foot, may be lost. Later the grown girl, with woefully impeded circulation in her feet, suffers from such extreme forms of chilblains and inflammatory troubles that it is not surprising to find her slowly learning the habits of her Western sisters and refusing the process altogether. Nevertheless, the foot of a Chinese woman still remains to her a possession of the utmost importance. Amputation is strenuously resisted by the patient's friends.

